# Ultrafast strong-field terahertz nonlinear nanometasurfaces

**DOI:** 10.1515/nanoph-2022-0766

**Published:** 2023-02-15

**Authors:** Jiahua Cai, Sai Chen, Chunyan Geng, Jianghao Li, Baogang Quan, Xiaojun Wu

**Affiliations:** School of Electronic and Information Engineering, Beihang University, Beijing 100191, China; Zhangjiang Laboratory, Shanghai 201204, China; Beijing National Laboratory for Condensed Matter Physics, Institute of Physics, Chinese Academy of Sciences, Beijing 100190, China; Songshan Lake Material Laboratory, Dongguan, Guangdong 523808, China; University of Chinese Academy of Sciences, Beijing 100049, China

**Keywords:** impact ionization, intervalley scattering, photodoping, THz-pump THz-probe

## Abstract

Strong-field terahertz (THz)–matter interaction permits the investigation of nonequilibrium behaviors in the nonperturbative zone. However, the unavailability of a high-field free-space THz source with high repetition rates, excellent beam quality, and high stability hinders its development. In this work, we obtain the nonlinear modulation dynamics of a “THz-nano” metasurface on silicon substrates using a time-resolved strong-field THz-pump THz-probe (TPTP) with a thousand orders local field enhancement through confining THz waves into nano-gaps (15 nm, *λ*/33,000). By switching the THz field strength, we successfully realize a self-modulation ∼50 GHz frequency shift, which is further verified via the TPTP ultrafast time-resolution technique. The phenomenon is attributed to the impact ionization (IMI) of the silicon substrate under the excitation of extremely confined strong THz fields in nano-gaps. Both strong-field induced intervalley scattering (IVS) and IMI effects of photodoped silicon occurring in nano-gaps and large-area substrates were also observed by 800 nm optical injection of carriers. These aforementioned findings provide a robust research platform for the realization of ultrafast time resolution nanoscale strong-field THz–matter interaction and new ideas for nonextreme laboratories to realize extreme THz science, applications, and THz nonlinear modulation device development.

## 1 Introduction

THz waves are electromagnetic waves that have a frequency between 0.1 and 10 THz. The photon energy of THz waves matches the vibration and rotation energy levels of matter, making THz spectroscopy a crucial tool for physical information, material characterization, and biomedical applications [1–10]. Consequently, in the past two decades, numerous types of THz spectroscopy instruments, such as THz time-domain spectroscopy (THz-TDS) and frequency-domain spectroscopy (THz-FDS), have been rapidly developed. Particularly, THz-TDS have found widespread use in industrial environments, medicine and biomedicine, agriculture and food, national defense and security, and other domains. The majority of these applications rely on monitoring the linear response of THz–matter interactions during THz waves’ probing with low power (weak electric field, ∼V/cm) but a high signal-to-noise ratio. In contrast, if the peak electric field reaches around kV/cm or even MV/cm, which is comparable to the electric field intensity between semiconductor atoms, THz waves can induce nonequilibrium states through electromagnetic field action [11, 12]. Additionally, the sufficient electric field strength is also possible to induce phase transitions or manipulate spins (using its magnetic fields) [3, 13], [14], [15].

To provide a strong THz field, optical rectification utilizing semiconductors (such as ZnTe) [16] or organic crystals [17], lithium niobate (LN) crystal based on the tilted pulse front technique [18–20], wide aperture photoconductive antennas [21], spintronic THz emitters [22], and laser-plasma THz sources [23, 24] have been created. In the meantime, the pump-probe technique, which is frequently employed to assess the photogenerated excited state absorption, is introduced to study the nonequilibrium/nonlinear states under an intense THz pump. There have been advancements in TPTP, THz-pump optical-probe (TPOP), and THz-pump visible-magneto optic Kerr effect (MOKE) measurement to explore THz-induced IMI [25, 26], electron–phonon interaction in semiconductors [27], phase transitions in VO_2_ [13], etc. However, it still cannot satisfy the requirements for vital scientific and technological studies in nonlinear THz physics, which are anticipated to reach 1 MV/cm or even higher under the excitation of average femtosecond lasers. How to reach the level of the electric field without using incredibly strong pump laser sources becomes a formidable obstacle.

Recent advances in subwavelength electromagnetic metamaterials/metasurfaces offer a possible method for enhancing localized electric fields [28–30]. Nonlinear responses produced by IVS, IMI, and interband tunneling in semiconductors, as well as phase transition, have been recorded by tens of times local field augmentation through micro-gap–enabled split-ring resonators (SRRs) [31–33]. The appearance of THz-nano metasurfaces has increased local field enhancement, hence, reducing the demand for strong incident fields. Using this nano-gap SRRs metasurface, it is possible to identify field-induced carrier multiplication in a high-resistance silicon substrate with an incident field intensity of ∼100 kV/cm or even less [34]. In addition to resonant metasurfaces, metagratings offer a broadband field enhancement that is strongly dependent on the gap size (often produced in nanometers) and duty cycles [35, 36]. Luminescence in quantum dots has been observed by THz field-driven interdot charge transfer through this method [12, 37]. However, the ultrafast dynamics that happened in the nano-gap–enabled nonlinear THz metasurfaces have not yet been systematically investigated.

In this work, through a nanometer-scale gap, the locally enhanced THz electric field induces the IMI of the substrate to produce nonlinear effects. This has been observed by frequency self-modulation as in [34]. Using TPTP, we revealed that THz driving carrier dynamics in silicon. Through the ingenious design of the experimental scheme, at a pumping field intensity of 127 kV/cm, a frequency shift of 21 GHz is generated in the transmission spectrum. This non–self-modulation phenomenon is the direct evidence of carrier generation by THz field–induced IMI in the high resistance silicon substrate. During this period, we investigate the nonlinear effects in photodoped silicon substrates by introducing intense THz into optical-pump THz-probe (OPTP). With the increase of incident THz field strength, the THz field–matter interaction goes through two nonlinear phases, IVS in the gap (phase I) then IMI in the gap and IVS in the substrate (phase II). These findings characterize in full the ultrafast nonlinear THz physics in THz-nano metasurfaces and provide a new perspective for researching ultrafast electronics in semiconductors working in THz frequencies.

## 2 Integrated strong THz pump and multispectral probing time-domain system

**Figure 1:**
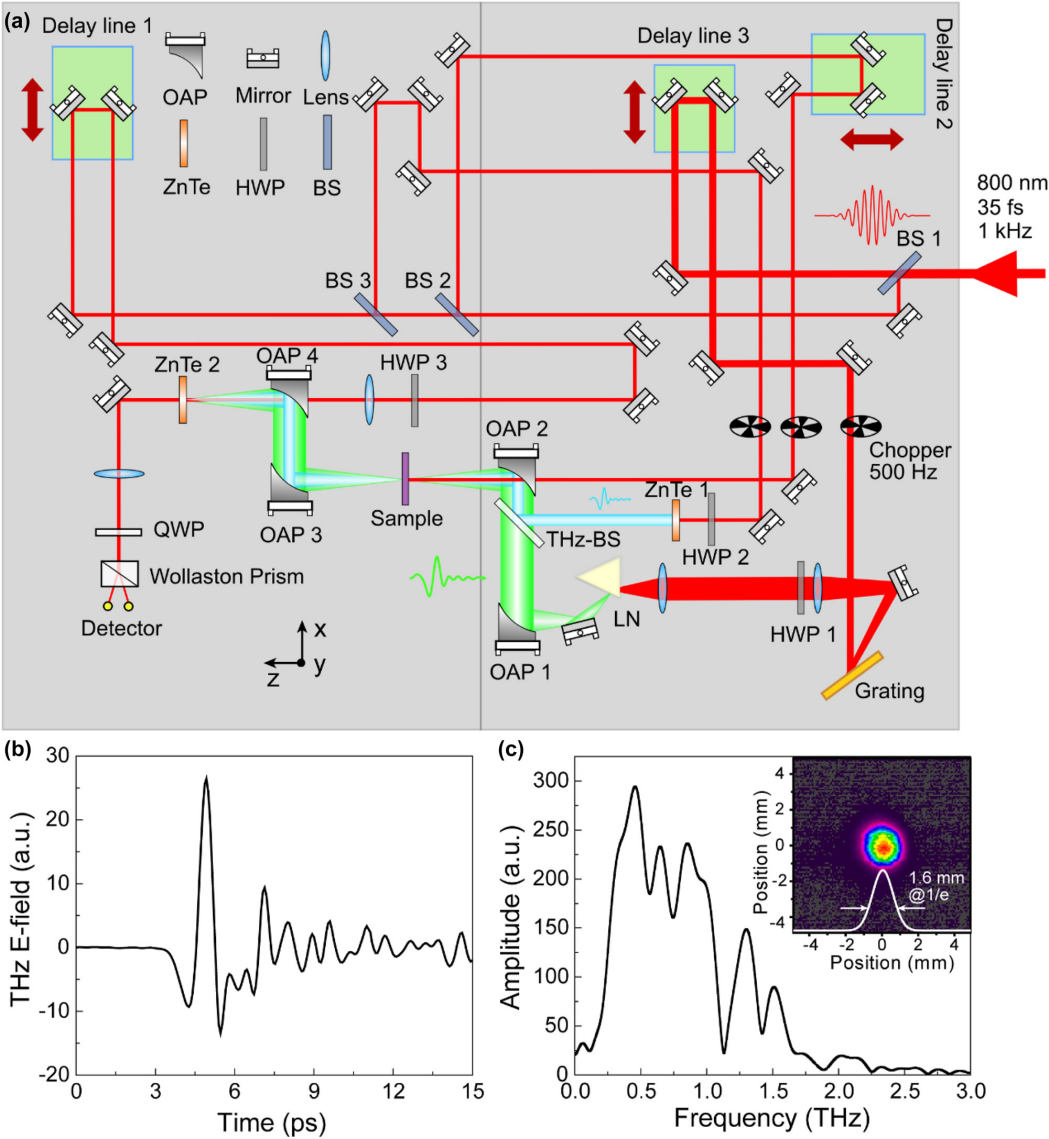
Integrated strong THz pump and multispectral probing time-domain system. (a) The schematic of the system. (b) The generated strong-field THz temporal waveform and (c) its corresponding spectrum. The inset in (c) shows the focused THz beam profile with a diameter of ≈1.6 mm (1/*e*) measured at the focus of OAP 2.

The experiments were implemented on our home-built–integrated strong-field THz pump and multiple spectral probing system. As illustrated in Figure 1, the laser output from a commercial Ti:sapphire laser amplifier is split by an 80:20 beam splitter. The transmitted beam (80%) is employed as a pump beam to generate strong-field THz from LN. After chopping, the pump beam is incident into the grating and its diffracted light is incident onto the LN crystal through two lenses (Lens1 and Lens2) and a half-wave plate (HWP) 1. The radiated THz pulses are collimated with an off-axis parabolic mirror (OAP) 1 and then focused on the sample by OAP 2. The transmitted THz signal is further collimated by OAP 3 and then focused by OAP 4 onto the ZnTe detection crystal (ZnTe 2). The residual 20% femtosecond laser energy is divided into three beams, one for photodoping, the second for generating weak-field THz probing beam in ZnTe emission crystal (ZnTe 1), and the third for the electro-optic sampling of both the pump and probing THz temporal waveforms. Three delay lines are employed to synchronize the strong-field THz, optical pump, and electro-optic sampling. This flexible integrated multispectral THz system is equipped with strong-field THz nonlinear spectroscopy, TPTP, and OPTP capabilities, among others. The generated strong-field THz temporal waveform and its corresponding spectrum are illustrated in Figure 1(b) and (c). The THz pump has a pulse duration of ∼0.75 ps, with a peak field located at 0.45 THz. The focused THz single pulse energy is measured by a calibrated pyroelectric detector (SDX-1152, Gentec) and its maximum THz energy used in our experiment is 4.8 µJ. The focused THz profile is exhibited in the inset of Figure 1(c), which is recorded by a commercial THz camera. With the focused THz maximum single pulse energy as well as its beam size, the calculated peak electric field at the focus of OAP 2 can reach 350 kV/cm. All the measurements are conducted in ambient air. In Figure 1, we also define the propagation of the strong THz pulse at the sample position as axis *z*, the polarization of its electric field as axis *y*, and the polarization of its magnetic field as axis *x*. See Supplementary Material for more details about the setup as well as the weak-field THz probing information.

**Figure 2:**
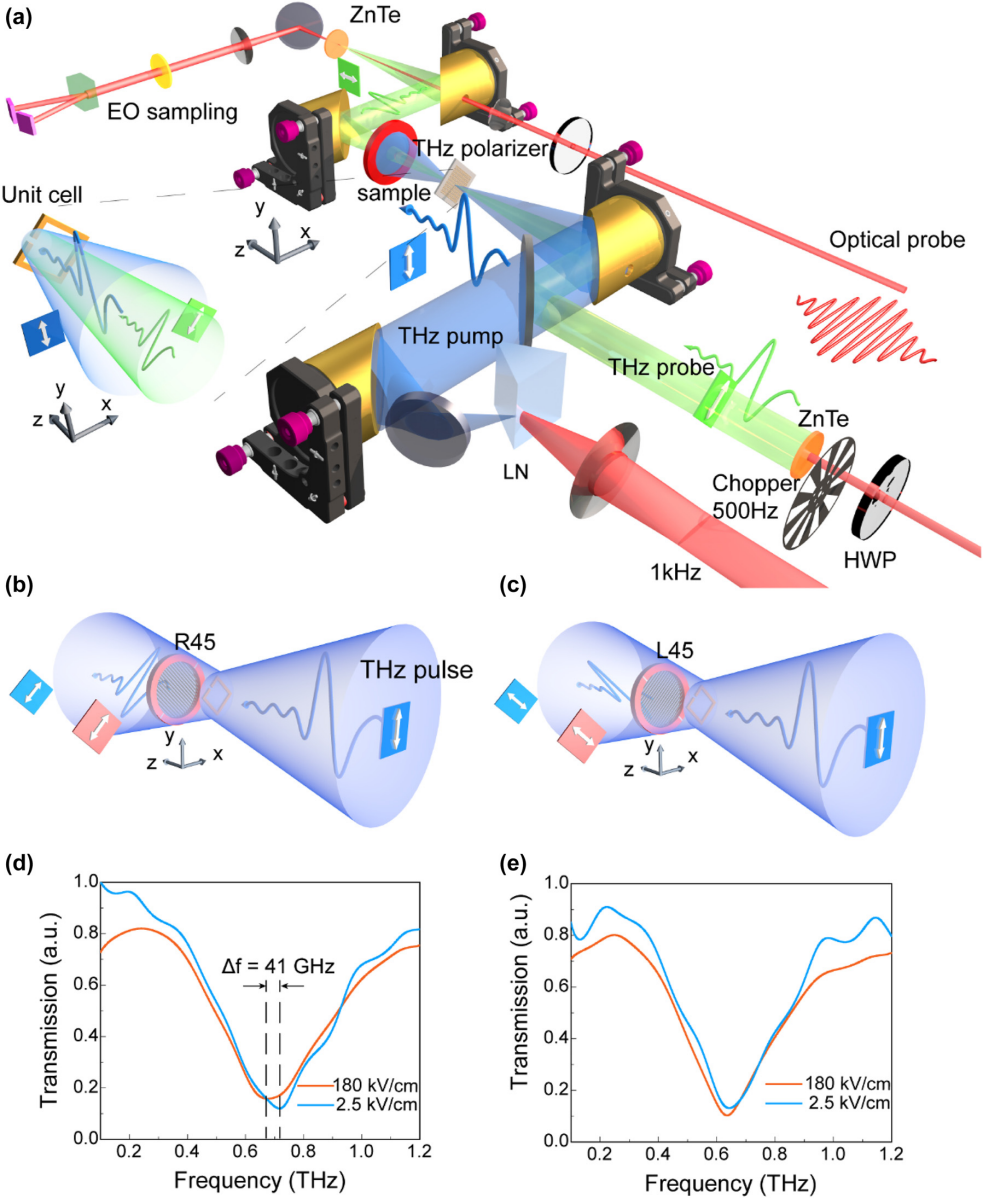
Carrier dynamics in the nano-gap via TPTP. (a) Schematic of the enlarged TPTP function. (b) and (c) Local schematic of the optical path setup for the validation experiment. (b) The sample is rotated by 45° along the *z*-axis, and a THz polarizer with the polarizing direction of 45° is placed behind the sample, allowing only the transmission of THz waves in TM polarization; (c) the polarizing direction of the THz polarizer is −45°, and only the THz waves in the TE polarization can transmit. (d) The TM polarization transmission spectra measured at the incident fields of 2.5 and 180 kV/cm in Figure 2(b), showing a clear nonlinear frequency modulation. (e) Corresponding TE polarization transmission spectra in Figure 2(c), showing no significant nonlinear frequency modulation. The combination of Supplementary Material shows that this setting can measure nonlinear frequency modulation and the necessity of using the THz polarizer.

The THz-nano metasurfaces measured in the experiment consist of a square array of gold SRRs, whose unit cell is illustrated in Figure 2(a). The gold ring cell has a square unit period of 69 μm with a center length of 47 μm, an arms width of 6 μm, and a thickness of 80 nm. A nano-gap width of *g* = 15 nm is fabricated at the center of one arm, and the substrate is a 0.5-mm thick high-resistance silicon wafer. Using conventional photolithography techniques and the sample tilting method, the large-scale metasurfaces were fabricated. Without the need for focused ion beam (FIB) and electron beam lithography (EBL), this smart approach enables us to achieve 15-nm gaps in micron-sized SRR array over a surface area of 1.5 cm × 1.5 cm. For more details on sample preparation and characterization, see [34]. For the electric field along the arm with a gap (TM polarization), it is calculated by numerical simulation that there are about 3 orders of magnitude of local field enhancement effects at the nano-gap.

## 3 Carrier dynamics in nano-gaps investigated by TPTP

To verify the performance of the sample and the strong-field THz-induced nonlinearity in these nanometasurfaces, we first investigate the nonlinear frequency modulation behavior via varying the illuminated THz field strengths, while probing the transmitted self-modulated strong-field THz itself in the meantime. The TM and TE polarizations correspond to the direction of the THz electric field parallel and perpendicular to the arm containing the nano-gap, respectively. For the strong-field THz-induced nonlinearity observation, we detect a nonlinear response under TM polarization for the sample including the THz-nano metasurface. When the THz electric field is increased from 2.5 kV/cm to 180 kV/cm, the resonant frequency lowers from 0.73 THz to 0.68 THz, resulting in a nonlinear frequency redshift of approximately 50 GHz. This phenomenon is similar to our previous results [34], indicating the effectiveness of the metasurface used in the experiment. More details are shown in the Supplementary Material.

Then, we employ the TPTP technique to capture the frequency shift phenomenon by introducing a weak-field THz probing beam, to investigate the dynamic mechanism of the nonlinear frequency modulation generated by the strong-field THz. Figure 2(a) depicts a 3D enlarged illustration of the TPTP experimental setup. With a field strength of 180 kV/cm for the THz pump beam, its polarization direction is perpendicular to the horizontal plane. The polarization direction of the weak-field THz probing beam generated from the ZnTe 1 can be adjusted by a half-wave plate in the excitation 800-nm femtosecond laser beam. The THz probing beam polarization is set at a 45° angle concerning that of the THz pump beam. Coaxially aligning two THz waves (for pumping and probing) and then focusing them onto the sample with an OAP mirror. To obtain the pure probing signal, the THz probing beam is modulated by a chopper with a 500 Hz rotor frequency. In addition, a THz polarizer is also positioned behind the sample with its polarizing orientation perpendicular to the THz pump to further restrict the transmitted THz pump from entering the detection module.

By this configuration, the samples are additionally rotated by 45° along the *z*-axis to match the TM or TE mode of the THz probing beam. In this configuration, the highest equivalent electric field of the pumping waves used for nonlinear modulation is the square root of that stated in the previous paragraph (127 kV/cm). In this situation, it is necessary to assess whether the THz pump can still trigger nonlinear frequency modulation. Figure 2(b) and (c) depict the experimental setup for the verification experiment. At this point, the THz probe is blocked, and the THz polarizer behind the sample is utilized to extract the THz signal with polarization orthogonal (marked as R45) or parallel (marked as L45) to the SRRs nano-gap. Under the incident field strength of 2.5 kV/cm and 180 kV/cm, the transmission of two cases (R45 and L45) are shown in Figure.2(d) and (e), respectively. Similar to previously reported findings, nonlinear frequency modulation (∼41 GHz) occurs in R45 but not in L45. This demonstrates that the THz pump can still achieve nonlinear frequency modulation following the 45° *z*-axis rotation of the SRRs.

**Figure 3:**
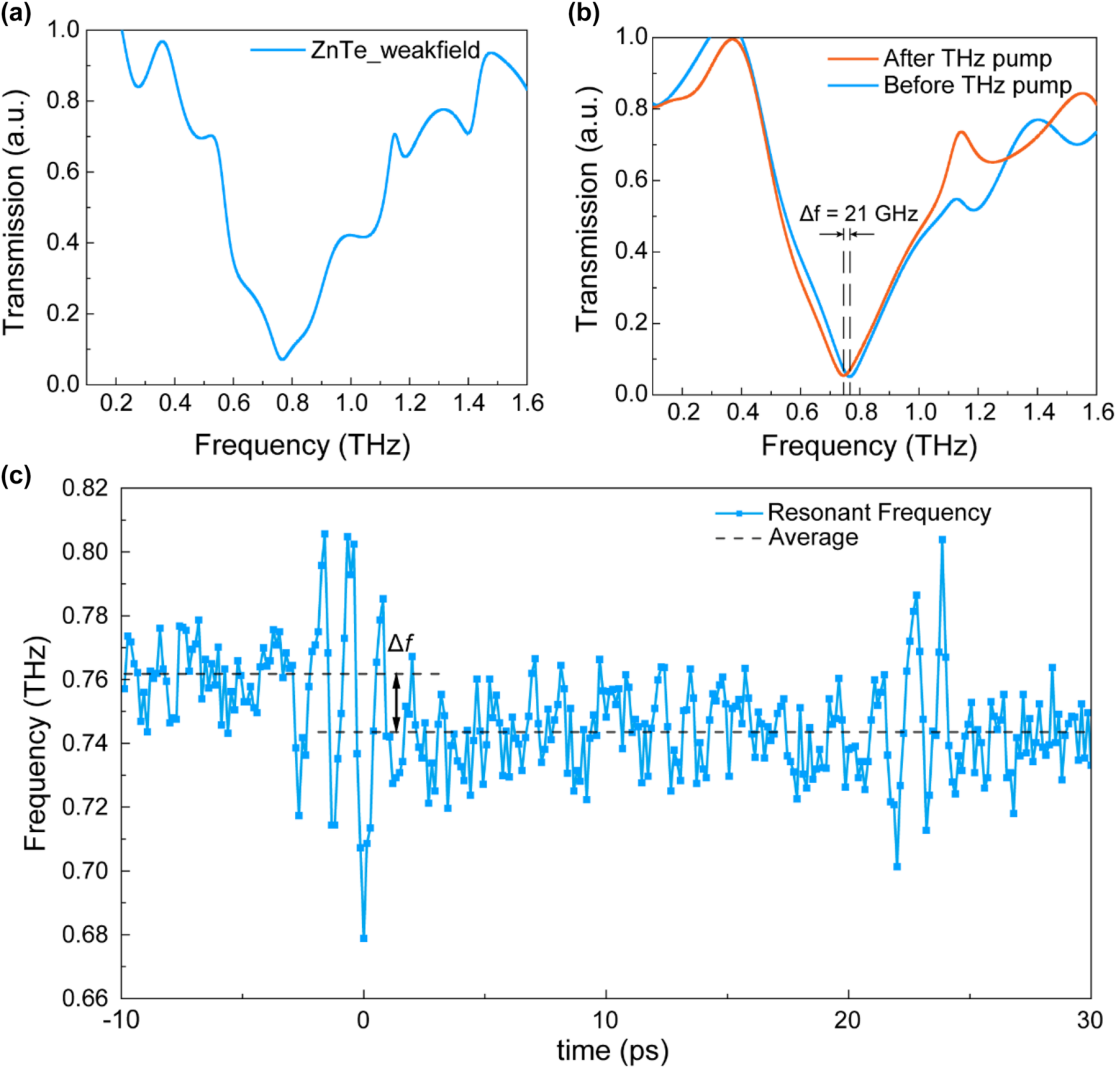
TPTP of THz-nano metasurface. (a) The transmission spectra of the THz probe with the resonance frequency at 0.764 THz. (b) Typical THz probe transmission spectra before and after the THz pump, showing a frequency shift of 21 GHz. (c) TPTP dynamic curve of resonant frequency-time delay for the THz-nano metasurface on the highly resistive silicon substrate.

During TPTP measurement, the horizontal component of the probing THz waves propagating through the polarizer can be observed by electro-optic sampling. Figure 3(a) illustrates the transmission spectra of the SRRs sample from the THz probe without an intense THz pump, which displays a resonance at 0.764 THz. As shown in Figure 3(b), before (at −5.2 ps) and after (at 16.67 ps) intense THz pumping, the resonance frequency is 0.766 THz and 0.745 THz, respectively. It can be concluded that, after intense THz pumping, the resonance frequency shifts by 21 GHz. The dynamic shift of the resonant frequency in the time domain is depicted in Figure 3(c). The blue solid line corresponds to the experimental data, whereas the black dashed line indicates the average value of experimental data before or after intense THz pumping. Within the measuring period, the shift of the resonance frequency from 0.762 THz to 0.744 THz (based on the average value) recovers only a little. This is because the carrier lifetime of high-resistance silicon is at least 10 μs, which is significantly longer than the measurement period (∼50 ps). This phenomenon is direct evidence that carrier production in this SRRs sample is caused by the IMI of high-resistance silicon.

## 4 Nonlinear response induced by both photodoping and THz field

In this part, we examine the nonlinear response of THz transmission spectra when an optical pump is introduced, while the strong-field THz as both pump and probe (it can also be treated as self-modulation when the weak-field THz from ZnTe 1 is blocked). Here, we regard the optical pump as a doping source and consider the influence of substrate initial conductivity varying caused by the optical pump on the subsequent THz field–substrate nonlinear interaction. We find the mechanism of nonlinear response induced by photodoping and THz field, which shows different physical processes under varying incident THz field strength. This provides a more systematic explanation for the phenomenon of difference in the nonlinear response obtained by using only two different intensity THz probes in [34]. The silicon substrate is photodoped using an 800-nm femtosecond laser with a pump flow of 83 μJ/cm^2^ to excite photogenerated carriers. We estimate that the substrate surface conductivity in this case (see Supplementary Material) is 650 S/m. According to the Drude–Smith model, the conductivity of 650 S/m on the substrate surface corresponds to a doping concentration of approximately 1.3 × 10^19^ cm^−3^ in the example (see Supplementary Material). The time delay between the THz probe and the 800-nm pump is 46 ps, which is significantly less than the carrier lifetime of high-resistance silicon.

**Figure 4:**
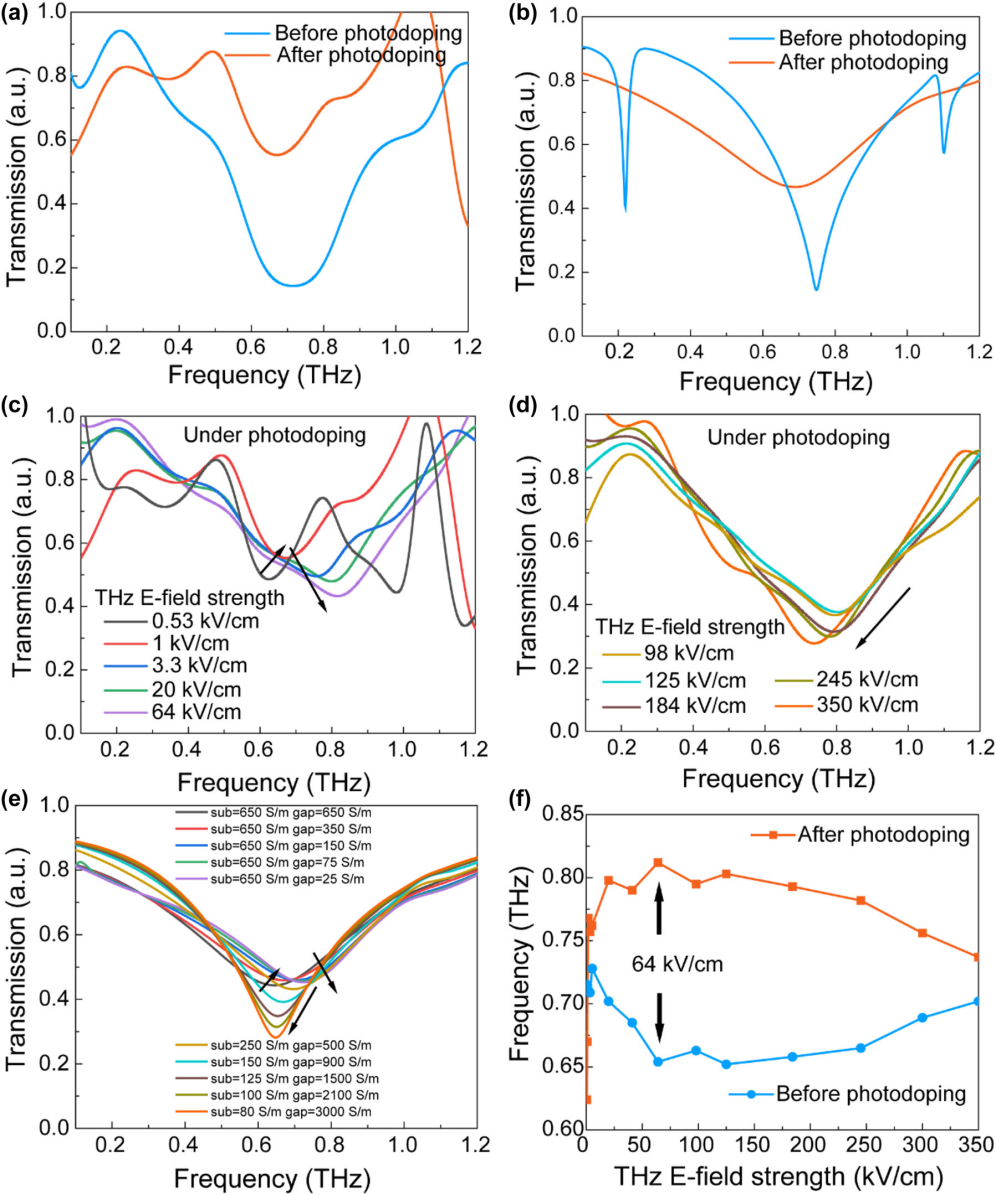
Nonlinear response of THz-nano metasurface induced by photodoping and THz field. (a) Photodoping induced transmission nonlinearity under an incident THz probe field strength of 1 kV/cm and (b) its numerical simulation results. (c) THz transmission with incident field strength ≤ 64 kV/cm. (d) THz transmission of 64 kV/cm ≤ incident field strength ≤ 350 kV/cm. (e) Numerical simulation results corresponding to (c) and (d). (f) The resonance frequency variation before and after 800-nm laser photodoping as a function of the incident THz field strength.

The experiment results and numerical simulation are shown in Figure 4(a) and (b), respectively. The photon energy of the 800-nm near-infrared pump pulse (1.55 eV) is greater than the band gap of intrinsic silicon (1.12 eV) at room temperature. After the sample is excited, the electrons in the valence band are excited to the conduction band, resulting in electron–hole pairs. The increased carrier concentration increases the conductivity of the substrate to 650 S/m. The THz field then interacts with the photodoped substrate. In the case of high conductivity, the sample shows low transmission and weak resonance. When the incident field strength of the THz probe is increasing from 0.53 kV/cm to 64 kV/cm, the transmission spectra after photodoping reveal a blueshift of the resonance frequency as depicted in Figure 4(c). This physical process can be explained by the IVS effect of electrons in the conduction band of doped silicon. The electrons in the low energy valley of the conduction band are excited to the high energy valley by strong field THz, and the electrons occupying the high energy valley have greater effective mass, leading to reduced mobility and conductivity. Note that the off-resonance transmission remains almost constant at lower frequencies (<0.3 THz), implying that the reduction in conductivity due to IVS is only localized in the nano-gap. We are positive that the IVS that occurred close to the nano-gap was a result of the strong electric field. The decrease in conductivity coincides with the opening of the nano-gap, causing a blueshift in the resonance frequency. The numerical simulation is displayed in Figure 4(e), in which we modify the conductivity in the nano-gap to decrease from 650 S/m to 25 S/m, revealing similar trends.

However, as illustrated in Figure 4(d), as the incident field strength of the THz probe continues to increase, the photodoping-induced frequency shift switches from a blueshift to a redshift. As the incident field strength grows, the resonance intensity remains elevated, the resonance frequency begins to red shift, and the off-resonance transmission increases at lower frequencies. This suggests an increase in conductivity at the nano-gap and a decrease in conductivity across the substrate. We also measure the transmission spectra of a bare photodoped high-resistance silicon substrate under various incident THz fields (see Supplementary Material) and demonstrate that the transmission gradually increases when the incident field is greater than 64 kV/cm, indicating the presence of IVS. However, as a result of the extraordinarily strong local field generated by the enhancement of the nano-gap, IMI and conductivity rise, the nano-gap closes, and the resonance frequency is red shifted. This physical process can be viewed as the substrate conductivity beginning to diminish while that of the nano-gap continues to rise. Therefore, in the simulation, we cause the substrate surface conductivity to decline from 250 S/m to 80 S/m and the gap to expand from 500 S/m to 3000 S/m, and the trend is strikingly similar to the experimental data. Note that the simulated findings and experimental outcomes are not identical. It is possible that the Gaussian distribution of the THz spot contributes to varying field strengths at various SRRs, causing the conductivity of the substrate and nano-gap to differ with position, and that this complex conductivity distribution accounts for the deviation from the ideal results.

Figure 4(f) illustrates the extracted resonance frequency before and after the 800-nm laser photodoping with the variation of incident THz field strengths. The dependence of both resonance frequencies on field strength turns at 64 kV/cm. For the case after photodoping as described previously. However, before (without) photodoping shows a completely different field intensity dependence process from that after photodoping. The resonance frequency remains stable at a low field (≤5.3 kV/cm), and then starts to redshift, the field strength of 64 kV/cm already reached the lowest resonance frequency, allowing the closure of the nano-gap. With further improvement of THz field peak strength, the resonant frequency shifts blue, which we think is because the field strength before reaching the peak value of the THz pulse is enough to induce IMI at the nanogap. With the further increased field strength, IVS at the nanogap is induced with the existence of the carriers generated by IMI, making the gap open and the frequency blueshifts. That is, a single THz pulse continuously induces two processes, IMI and IVS at the nano-gap. This phenomenon needs to be investigated further and more detailed. This contrast highlights the significant changes induced by the optical pump. In the future, it will be an interesting topic to study the field strength-dependent convolute response under different doping concentrations.

## 5 Conclusions

In summary, we demonstrate a new class of reconfigurable “THz-nano” nonlinear metamaterial that shows remarkable field-induced nonlinear self-modulation of resonances at THz frequencies through strong-field–induced carrier multiplication effects. The developed strong-field THz pump-multiple spectroscopy probing system enables the capability of the observation of ultrafast carrier dynamics in the 15-nm nano-gap in large-area micron-scale structures. The investigation of competitive physical effects from nonlinear THz field-induced IVS, IMI, and the interband excitations in silicon using the ultrafast THz/optical pump and intense/weak THz probe spectroscopy. The fabricated device consists of nano-gaps (*λ*/33,000; 15 nm), with strong enhancement of localized THz fields in an extremely confined spatial region, thereby providing a unique way of probing THz nonlinearity at the nanoscale and achieving strong nonlinearity without the need for intense sources. Our work may open a way to the development of photodetectors, lab-on-a-chip integrated (bio)sensors, and ultrafast switching based on THz-nano metasurfaces. In addition, at the same gap scale, by using the metasurface unit cell based on THz asymmetric split-ring resonator (TASR) and the strong field THz-metamaterial–background material coupling resonance to modulate the Fano resonance intensity [38, 39], a new all-THz ultrafast device may be realized.

## Supplementary Material

Supplementary Material Details
