# Supplementary material for: Ultrafast strong-field terahertz nonlinear nanometasurfaces
Source: Nanophotonics. 2023 Feb 15;12(13):2517–26. doi: 10.1515/nanoph-2022-0766 (PMC11501971; doi:10.1515/nanoph-2022-0766)
Supplement: Supplementary file 1 — Supplementary Material Details [file j_nanoph-2022-0766_suppl.docx]

Supplementary Material for

**Ultrafast Strong-Field Terahertz Nonlinear Nanometasurfaces**

Jiahua Cai, Sai Chen, Chunyan Geng, Jianghao Li, Baogang Quan, and Xiaojun Wu

**1. The Experimental Setup**

The strong-field THz pump and multiple spectral probing system we built is illustrated in Figure 1. We employ a Ti: sapphire femtosecond laser amplifier, which can provide laser pulses with a center frequency of 800 nm, pulse duration of 35 fs, a repetition rate of 1 kHz, and maximum pump power of 5 mJ. The strong THz pulse in the system is generated by LN crystal through optical rectification based on the tilted pulse front technique.

Detection of THz temporal waveforms is performed by an electro-optic sampling system consisting of a ZnTe crystal, a quarter-wave plate, a Wollaston prism and two photodiodes for coherent detection of THz pulses through the principle of electro-optic effect. To realize the functions of OPTP and TPTP, three delay lines are added. Delay line 1 (DL1) for THz electric field coherence detection, delay line 2 (DL2) for controlling the time delay between the 800 nm optical pump and THz probe in the OPTP system, and delay line 3 (DL3) for controlling the time delay in the TPTP system.

The generated typical strong-field THz temporal waveform and its corresponding Fourier transform spectrum are depicted in Figure S1 (a) and (b), respectively. At the focus of OAP2, the diameter of the beam profile is 1.6 mm (1/*e*) and has a Gaussian beam distribution. Based on the focused THz single pulse energy and its beam profile, we can use the following formula to calculate the focused free-space THz peak electric field[1],

$E_{0}\text{=}\sqrt{\frac{W}{c\varepsilon_{0}A\tau}}$ (1)

where *W* is the THz pulse energy, *c* is the speed of light in vacuum, *ε*_0_ is vacuum permittivity, the normalized spatial field profile *E*(*x*,*y*) and temporal field profile *E*(*t*), *A* and *τ* is given by,

$A=\int_{x} \int_{y} \left|E(x,y)\right|^{2}dxdy$ (2)

$\tau=\int_{t} \left|E(t)\right|^{2}dt$ (3)

For a Gaussian shape THz spot, *A* can be calculated as *πa*^2^, where *a* is the 1/*e* beam radius. In this case, *a* is equal to 0.8 mm. Based on the normalized THz waveform, *τ* is equal to 0.75 ps. Therefore, the calculated maximum THz peak electric field of 350 kV/cm.

The temporal waveform of the weak-field THz probe and its corresponding spectrum are illustrated in Figure S1 (c) and (d), respectively. When comparing its peak electric field with that of the strong-field from lithium niobate crystal, the weak-field THz from ZnTe is ~1/100 of the strong-field THz pump. Moreover, the peak frequency of the weak-field THz probe is located around ~1.0 THz, and its frequency range can reach up to 2.5 THz. These absorption peaks come from the interference of water vapor.


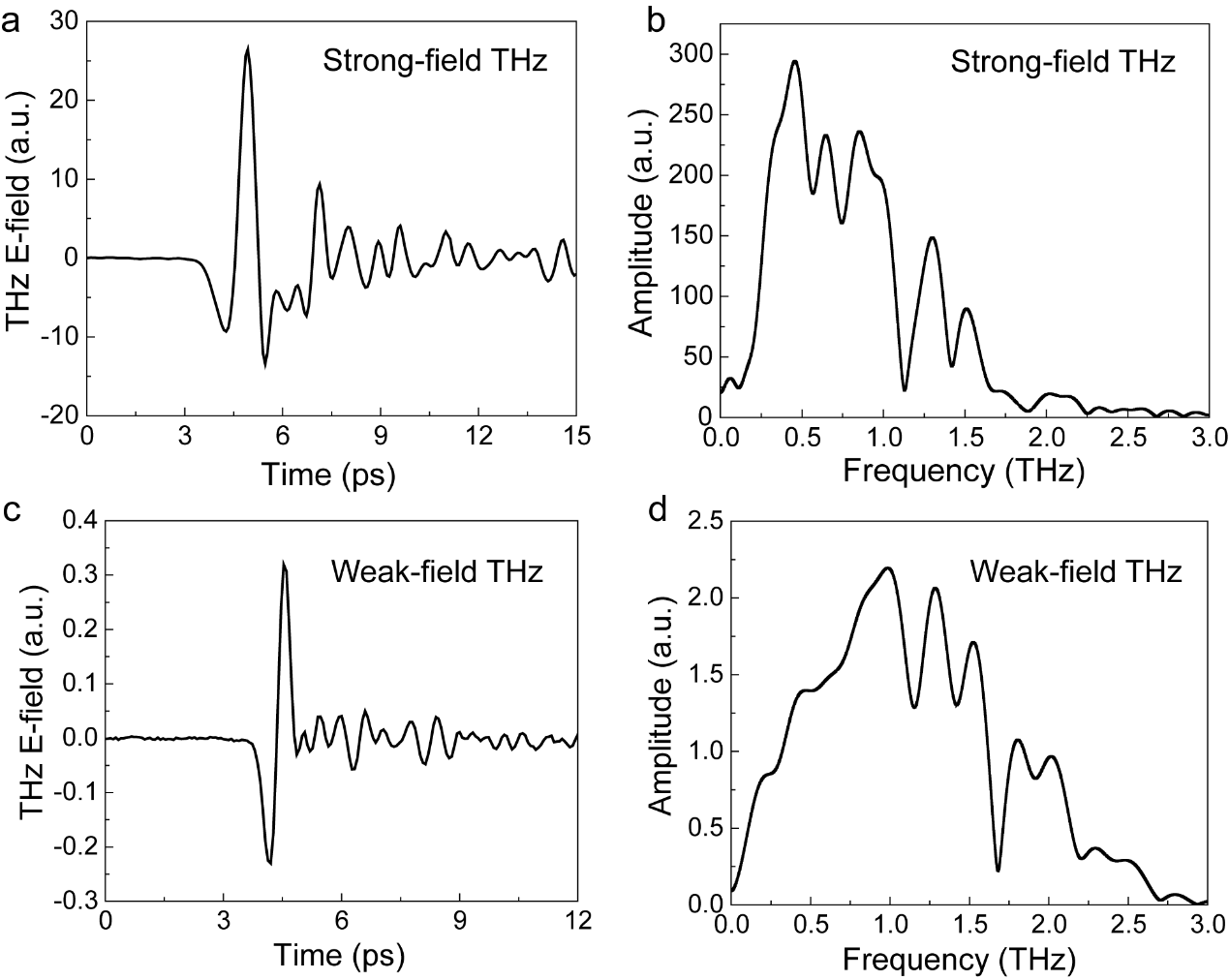


Figure S1: The temporal waveform of (a) the strong-field THz from lithium niobate, (c) the weak-field THz probe from ZnTe, and their corresponding spectra for (b) for strong-field and (d) for weak-field, respectively.

**2. Nonlinear Frequency Modulation induced by Strong-Field THz**

The relationship between the incident electric field of TE and TM polarization and the orientation of the metasurface unit cell is shown in Figure S2 (a). Under TM polarization, when the THz electric field is increased from 2.5 kV/cm to 180 kV/cm, as shown in Figure S2 (b), the resonance frequency of the transmission field decreases from 0.73 THz to 0.68 THz. This nonlinear frequency self-modulation is caused by the carrier multiplication induced by cascade IMI excited by strong locally enhanced THz field enhancement in the nano-gap of the metasurface. When the field strength is 2.5 kV/cm, the nano-gap in the gold ring can be treated as dielectric insulation (air). Upon increasing the THz strength to 180 kV/cm, the enhanced THz electric field in the nano-gap induces cascade impact ionization, leading to producing carrier multiplication effect. Therefore, the conductivity of the silicon substrate under the nano-gap area is effectively enhanced and closes the nano-gap, resulting in a resonant frequency shift. The corresponding simulation results by numerical calculation are shown in Figure S2(c). The incident field for 2.5 kV/cm corresponds to an “OFF” state at the gap (conductivity=0 S/m) and 180 kV/cm corresponds to an “ON” state (conductivity=5×10^5^ S/m). By the experiment results, the simulation implies that the opening and shutting of the nano-gap causes a nonlinear modulation of the resonant frequency. Note that the frequency shift obtained from the experiment is smaller than the simulation result, we discuss the difference in section 5 of the main text. Regarding the TE polarization depicted in Figure S2(d), the modification of the THz field strength has a negligible effect on the resonance frequency, which is kept at 0.65 THz.


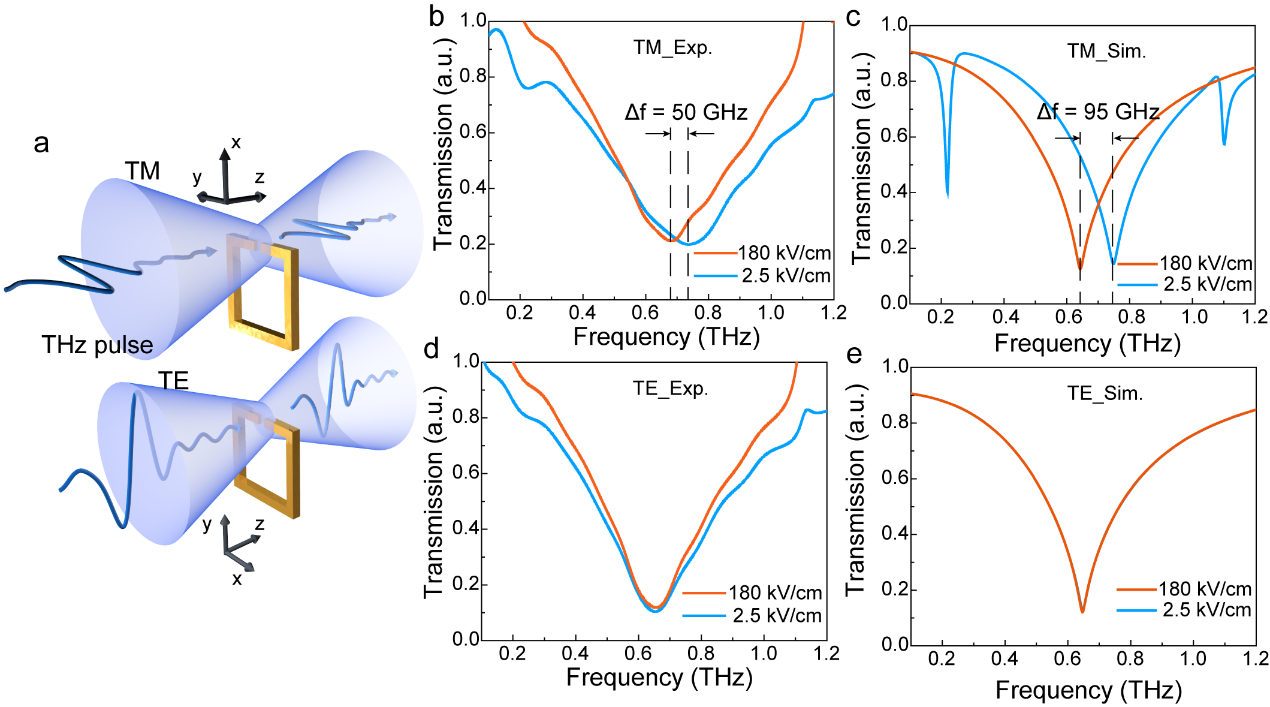


Figure S2: Strong-field THz induced nonlinear frequency modulation. (a) Schematic diagram of two different polarizations (TM and TE) of the incident THz pulse. (b) Measured transmission spectra under TM polarization at the incident fields of 2.5 and 180 kV/cm, showing a frequency shift of 50 GHz and its corresponding (c) simulation results calculated by the commercially CST Microwave Studio in the “OFF” (2.5 kV/cm) and “ON” (180 kV/cm) states of the nanogap. (dc) Experiments under TE polarization, not showing an obvious frequency shift. and its corresponding (e) simulation results, not showing an obvious frequency shift.

**3. THz polarizer for separating TM and TE polarization**

As mentioned in Section 4 of the main text, a THz polarizer can successfully differentiate between TM and TE polarizations when the SRRs are rotated 45° along the z-axis. Without the THz polarizer, the TM and TE polarization data are jumbled, resulting in a frequency shift of less than 10 GHz for incident field strengths between 2.5 kV/cm and 180 kV/cm, making it difficult to evaluate the nonlinear effects generated by the high THz field.


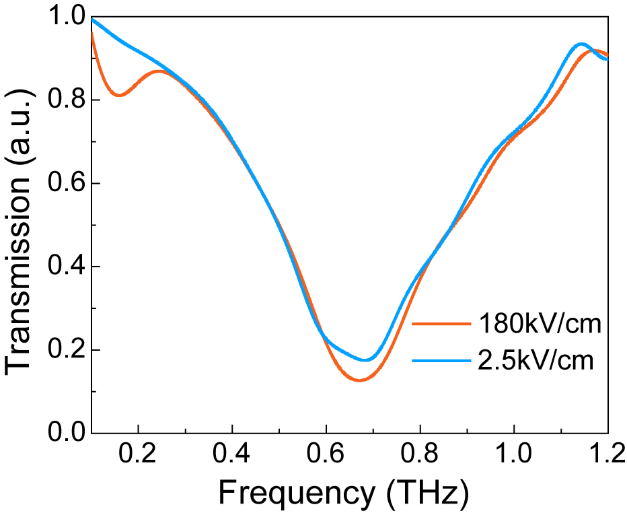


Figure S3: Transmission of THz-nano metasurface rotated by 45° along the z-axis without THz polarizer.

**4. Change in surface conductivity of the substrate generated by 800 nm laser photodoping**

The time-domain waveforms of the blank silicon substrate before and 46 ps after 800 nm laser photodoping at a pumping fluence of 83 μJ/cm^2^ and their Fourier transform spectra amplitudes are shown in Figure S4(a) and S4(b), respectively. The frequency-dependent complex conductivity after photodoping is measured by the THz transmission before and after photodoping. The ratio of the Fourier transform of its time-domain waveform is related to the complex conductivity $\widetilde{\sigma}(\omega)=\sigma_{1}(\omega)+i\sigma_{2}(\omega)$ [2, 3]:

$\frac{\widetilde{E}_{\text{before\_pump}}(\omega)}{\widetilde{E}_{\text{after\_pump}}(\omega)}=\frac{N+1}{N+1+Z_{0}d\widetilde{\sigma}(\omega)}$ (4)

where *N* is the refractive index of the unexcited silicon wafer, *Z*_0_ is the impedance of free space, and *d* is the photoexcited layer thickness[4]. The resulting calculated complex conductivity is illustrated as the dotted line in Figure S4(c). The real and imaginary parts of the conductivity are fitted using the Drude-Smith model [5]:

$\widetilde{\sigma}(\omega)=\frac{ne^{2}\tau/m^{*}}{(1-i\omega\tau)}[1+\sum_{j=1}^{\infty} \frac{c_{j}}{(1-i\omega\tau)^{j}}]$ (5)

The summation term in Eq.(5) truncates the part after the first term, *c*_1_ varies between –1 and 0 and is a measure of velocity persistence. For *c*_1_=0 is the Drude conductivity, and *c*_1_=–1 is the complete carriers’ backscattering or localization. *n* is the electron density, *e* is the elementary charge, and *τ* is the characteristic scattering time. *m** is the electron’s effective mass [5-7]. The fitting results are illustrated as the solid line in Figure S4(c). The agreement with the experimental results indicates that the experimental data are more consistent with the Drude-Smith theory.


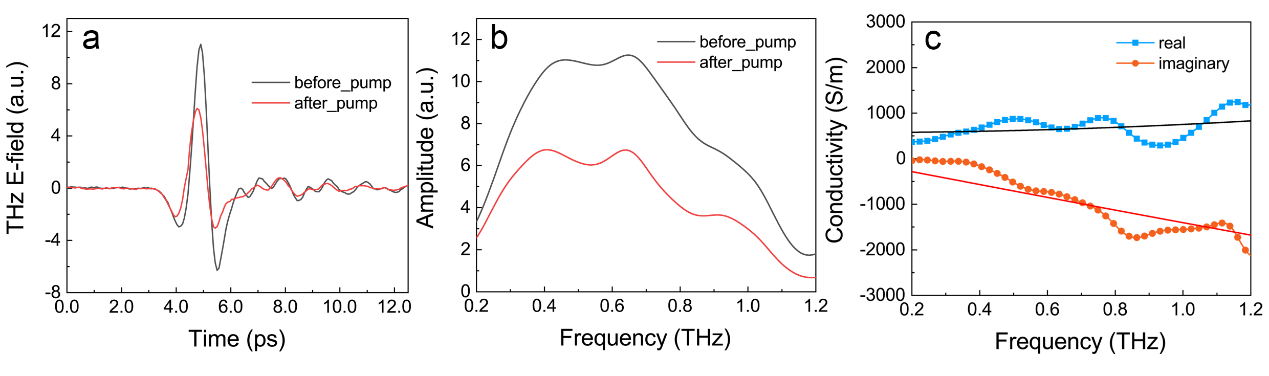


Figure S4: (a) The time-domain waveforms of the blank silicon substrate before and 46 ps after the optical pump and its (b) Fourier transform spectra amplitudes. (c) The calculated complex conductivity of the substrate surface after the optical pump and its corresponding Drude-Smith fitting results.

**5. Intervalley scattering of photodoped silicon substrate induced by strong THz field**

The results are depicted in Figure S5; we measured the transmission of the photodoped silicon substrate at various incident THz field intensities. After gradually increasing the incident field strength above 64 kV/cm, the photodoped silicon substrate's transmission begins to increase significantly and continues to increase with the incident field strength, indicating that the strong THz field induces intervalley scattering of the substrate, resulting in a decrease in its conductivity. Therefore, a field intensity of 64 kV/cm is regarded as the threshold for interval scattering to occur on a bare substrate.


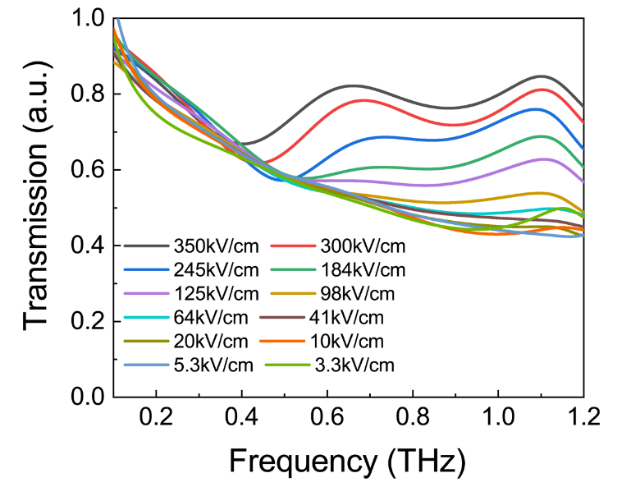


Figure S5: Transmission of photodoped silicon substrate under different incident THz field intensities.

Reference:

[1] B. L. Zhang, Z. Z. Ma, J. L. Ma, et al., "1.4-mJ High Energy Terahertz Radiation from Lithium Niobates," *Laser Photonics Rev*, vol. 15, no. 3, 2021.

[2] D. G. Cooke, F. A. Hegmann, E. C. Young, et al., "Electron mobility in dilute GaAs bismide and nitride alloys measured by time-resolved terahertz spectroscopy," *Appl. Phys. Lett.*, vol. 89, no. 12, 2006.

[3] M. Walther, D. G. Cooke, C. Sherstan, et al., "Terahertz conductivity of thin gold films at the metal-insulator percolation transition," *Physical Review B*, vol. 76, no. 12, 2007.

[4] G. F. Li, D. Li, Z. M. Jin, et al., "Photocarriers dynamics in silicon wafer studied with optical-pump terahertz-probe spectroscopy," *Opt. Commun.*, vol. 285, no. 20, pp. 4102-4106,2012.

[5] N. V. Smith, "Classical generalization of the Drude formula for the optical conductivity," *Physical Review B*, vol. 64, no. 15, 2001.

[6] Q. L. Zhou, Y. L. Shi, B. Jin, et al., "Ultrafast carrier dynamics and terahertz conductivity of photoexcited GaAs under electric field," *Appl. Phys. Lett.*, vol. 93, no. 10, 2008.

[7] A. T. Tarekegne, H. Hirori, K. Tanaka, et al., "Impact ionization dynamics in silicon by MV/cm THz fields," *New J Phys*, vol. 19, 2017.
